# Methylene Blue-Based Nano and Microparticles: Fabrication and Applications in Photodynamic Therapy

**DOI:** 10.3390/polym13223955

**Published:** 2021-11-16

**Authors:** Dong-Jin Lim

**Affiliations:** Department of Otolaryngology Head & Neck Surgery, University of Alabama at Birmingham, Birmingham, AL 35294-0012, USA; daniel.djlim@gmail.com

**Keywords:** methylene blue, photosensitizer, photodynamic therapy, antimicrobial photodynamic therapy

## Abstract

Methylene blue (MB) has been used in the textile industry since it was first extracted by the German chemist Heinrich Caro. Its pharmacological properties have also been applied toward the treatment of certain diseases such as methemoglobinemia, ifosfamide-induced encephalopathy, and thyroid conditions requiring surgery. Recently, the utilization of MB as a safe photosensitizer in photodynamic therapy (PDT) has received attention. Recent findings demonstrate that photoactivated MB exhibits not only anticancer activity but also antibacterial activity both in vitro and *in vivo*. However, due to the hydrophilic nature of MB, it is difficult to create MB-embedded nano- or microparticles capable of increasing the clinical efficacy of the PDT. This review aims to summarize fabrication techniques for MB-embedded nano and microparticles and to provide both in vitro and in vivo examples of MB-mediated PDT, thereby offering a future perspective on improving this promising clinical treatment modality. We also address examples of MB-mediated PDT in both cancer and infection treatments. Both in-vitro and in-vivo studies are summarized here to document recent trends in utilizing MB as an effective photosensitizer in PDT. Lastly, we discuss how developing efficient MB-carrying nano- and microparticle platforms would be able to increase the benefits of PDT.

## 1. Introduction

**Figure 1:**
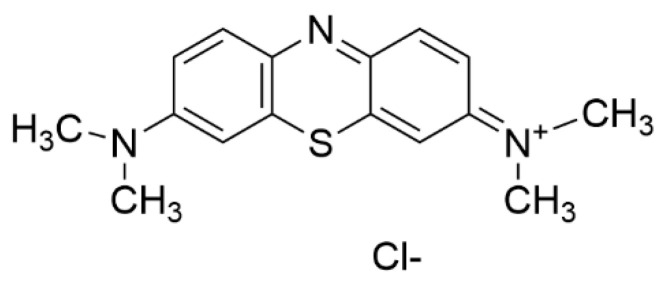
Chemical structure of methylene blue.

**Figure 2:**
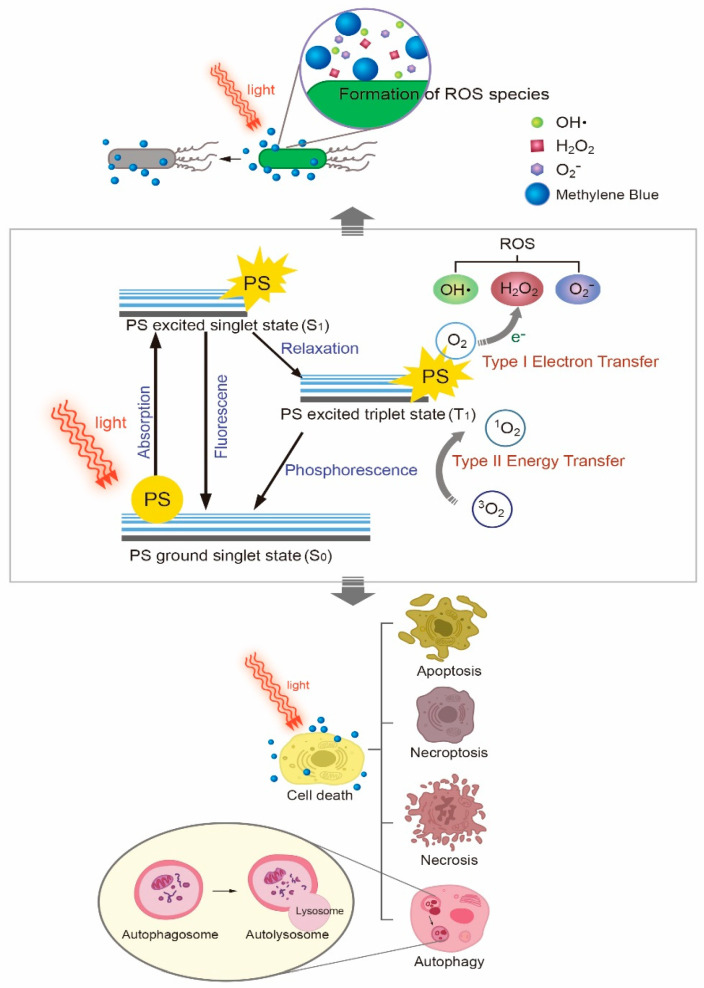
Type I and II reactions in photodynamic therapy and their influences on microbial cellular death and mammalian cell deaths. The types of mammalian cell death include apoptosis, necrosis, necroptosis, and autophagy.

Methylene blue (MB), first extracted by the German chemist Heinrich Caro, has been recognized not only as a dye, but also as a medicine that has been used in the treatment of malaria (Figure 1) [1]. MB can stain malarial parasites in infected red blood cells [2]. Thyroid surgery and the treatment of methemoglobinemia and ifosfamide-induced encephalopathy are a few typical examples of MB applications (Table 1) [3,4]. Preliminary investigations suggest that MB may also be used in the treatment of septic shock, neurodegenerative diseases including Alzheimer’s disease, and cancers [5,6,7,8,9,10,11]. When intravenously pretreated, MB can also reduce the pain associated with injection of propofol, which induces general anesthesia [12]. Moreover, MB is reported to show antiviral activity against COVID-19 caused by severe acute respiratory syndrome-coronavirus 2 (SARS-CoV-2) [13]. It hinders the binding interaction between SARS-CoV-2 spike protein and angiotensin-converting enzyme 2 (ACE2), which is known as the entry point of the virus. MB is commonly available in the market as a form of solution (e.g., 0.5% MB injection USP). The recommended dosage of MB is between 1 and 4 mg/kg, and oral and intravenous administrations for 5 to 10 min are suggested [14]. A terminal plasma half-life of MB after intravenous administration is about 5 to 7 h [15]. Because of the phenothiazinium chromophore of MB, it absorbs light near 630–680 nm, resulting in the formation of reactive oxygen species (ROS), including singlet oxygen [16,17]. Hence, MB is a photosensitizer essential for photodynamic therapy (PDT). PDT is a well-documented and evolving therapy wherein photosensitizing molecules are photoactivated and react with oxygen, thus helping to create ROS [18,19,20,21,22,23]. There has been growing interest in applying PDT for a variety of benign and malignant diseases [21,24,25]. For example, a light-excited photosensitizer (PS) such as MB generates cytotoxic reactive oxygen species (ROS) from molecular oxygen and achieves specific cancer cell death or tumor tissue damage [21]. The sensitized PS is able to create a superoxide anion radical (O_2_^•−^), a hydroxyl radical (OH^•^), or hydrogen peroxide (H_2_O_2_) through proton or electron transfer (type I mechanism) while the type II mechanism results in the formation of singlet oxygen (^1^O_2_) [26]. ROS produced by PDT is responsible for cancer cell death, and there are several PDT-mediated cell death pathways: apoptosis, necroptosis, autophagy, and necrosis. Details of the molecular mechanisms associated with them have been previously explained (Figure 2) [27,28]. Likewise, these oxidizing molecules also react with bacterial biomolecules, resulting in the eradication of infectious bacteria [29]. Antimicrobial photodynamic therapy (*a*PDT) uses the same principle of cytotoxic ROS formation under a specific wavelength of light and is recognized as a potential option for treating multidrug-resistance bacteria [30]. Several nanomaterials such as gold nanoparticles (NPs), silica NPs, and upconversion nanoparticles (UCNPs) have also been studied as PS carriers to improve the clinical potential of the PDT modality [31]. These materials improve the localized delivery of PS and enhance the efficacy of the irradiation of a specific light on the targeted tissue [32]. Moreover, they can protect the PS molecules from photobleaching [33].

The purpose of this review is to provide an overview of fabrication techniques for MB-embedded nano or microparticles and to share examples of MB-mediated PDT in the treatment of various cancers and infectious diseases in order to gain perspective on exploring new fabrication techniques for MB. MB-mediated PDT helps to combat against cancers and human infections through the cytotoxic effect of ROS, including singlet oxygen generated from photoactivated MB. Increasing the utilization of MB-embedded nano and microparticles in PDT may improve the clinical outcome of this promising therapy in the modern medicinal era.

## 2. Fabrication of Methylene Blue Nano and Microparticles

**Figure 3:**
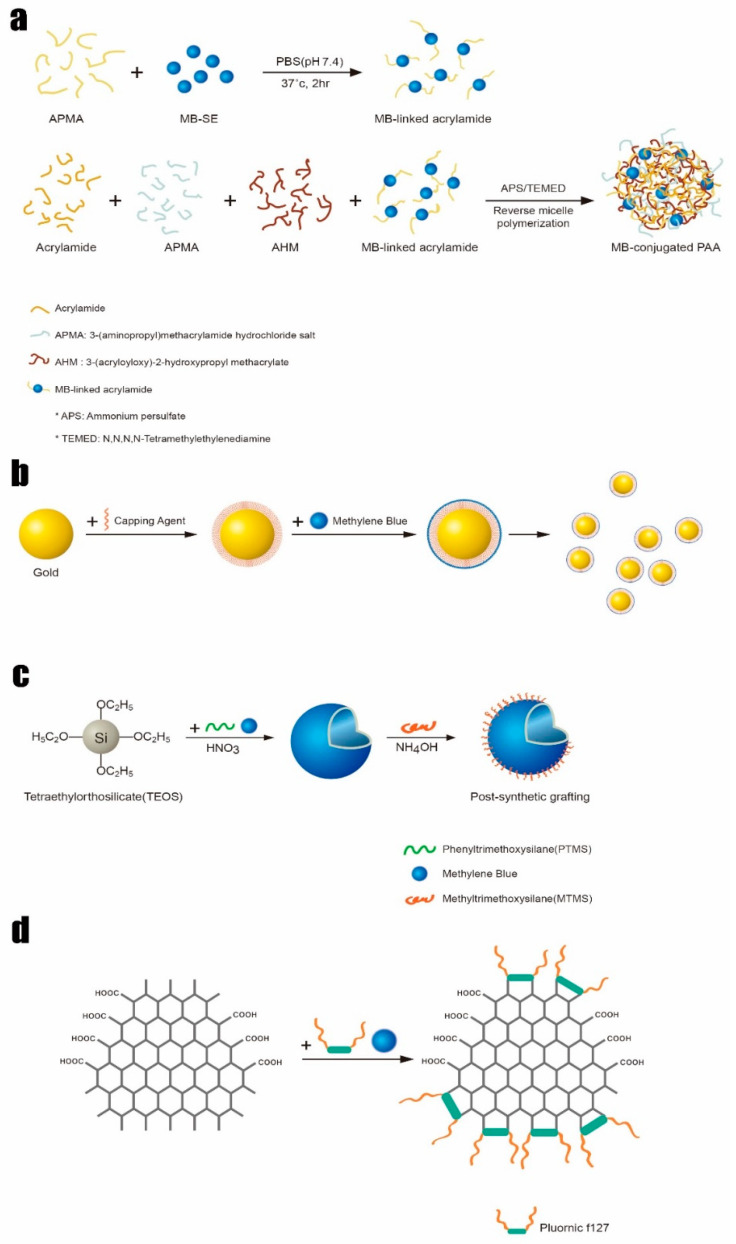
Fabrication techniques for methylene blue (MB) encapsulation. (**a**) MB-embedded polyacrylamide nanoparticles, (**b**) Gold nanoparticles electrostatically tethered with MB, (**c**) MB-embedded hollow silica nanoparticles, (**d**) Nano graphene oxide covalently tethered with MB.

Drug delivery through nano and microparticles is a recent trend for improving therapeutic outcomes while minimizing the side effects that are commonly involved with systemic drug delivery. A nano-sized or micro-sized PDT therapeutic tool has been considered as a promising modality that may have a number benefits [51]. Specifically, photosensitizer-embedded nano or microparticles can effectively deliver the photo-responsive molecules to tissues and cells and can even be used for translocating them into cellular compartments, thereby producing significant amounts of ROS in the target tissues. Targeted photosensitizer delivery would maximize the therapeutic efficacy of PDT while reducing immunogenicity and side effects. Several fabrication techniques for MB encapsulation have been studied (Figure 3) [52,53,54,55]. Polymeric nanoparticles, gold nanoparticles, silica nanoparticles, and nano graphene oxide as carbon-based nanomaterials have been utilized to improve MB-mediated PDT’s efficacy. This review presents both typical fabrication techniques and recent new strategies able to deliver MB in a sustained manner for improving the potential of MB-mediated PDT in clinical usages.

### 2.1. Methylene Blue in Polymeric Nano and Microparticles

MB can be incorporated into polymeric nanoparticles (NPs). In a 2005 study performed by Philbert group, MB was encapsulated within polyacrylamide NPs for the efficient delivery of singlet oxygen [52]. In this study, the MB-embedded polyacrylamide nanoparticles were fabricated by polymerizing acrylamide monomers and MB solution together. A 650 nm light stimulated the release of singlet oxygen from the entrapped MB, resulting in the damage of rat C6 glioma tumor cells, a model cancer cell line for glioblastoma. In the subsequent study, the polyacrylamide nanoparticles also retained the activity of embedded MB against diaphorase enzymes where the embedded MB within polyacrylamide NPs can enhance the therapeutic efficacy of PDT [53]. In another recent study, a poly (N-isopropylacrylamide) (PNIPAM) microgel was used to deliver MB without compromising its activity against enzymatic or environmental degradation [56]. This study showed that the PNIPAM microgel holds more MB than the PNIPAM-co-PAA microgel, whose outer surface consists of negatively charged PAAs (polyacrylic acids), indicating that the positively charged MB binds mostly to the AA groups located on the outer surface. To increase the loading efficacy, MB was covalently conjugated onto acrylamide in order to create MB-conjugated PAA nanoparticles, instead of physically entrapping MB in the polymeric matrix [57]. Using a tumor-homing peptide known as F3-peptide to provide a better affinity toward cancer cells, the authors demonstrated the possibility of using selective PDT for cancers. To achieve better singlet oxygen production in the NPs, a new strategy was studied where two different MB derivatives, 3,7-bisallylmethylene blue (MBI) and 3,7-bismethylacrylamide methylene blue (MBII), were synthesized and incorporated to create functionalized polyacrylamide nanoparticles [58].

**Figure 4:**
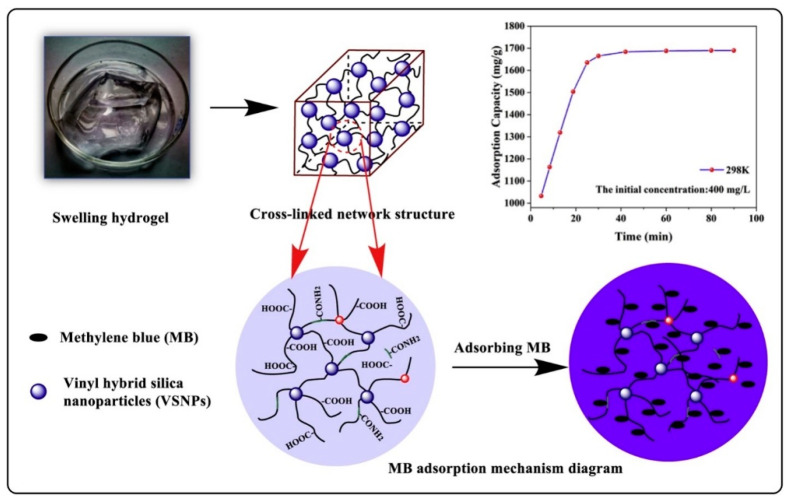
A novel super-adsorbent nanocomposite (NC) hydrogel adsorbent. In this study, a PAA [poly(acrylic acid)]-based hydrogel was prepared by vinyl hybrid silica nanoparticles (VSNPs) with average diameter of 30 nm as crosslinking agent. This adsorbent can absorb methylene blue at a high concentration (1690 mg/g) within 90 min. Reproduced from [59] with permission, copyright Elsevier, 2021.

A recent study demonstrated that MB can easily bind to the negative surface of nano-sized vinyl hybrid silica nanoparticles (VSNPs). Furthermore, when VSNPs were used as crosslinkers, the final PAA-based hydrogel can absorb significantly higher MB concentration than conventional PAA microgels. Although the purpose of this study was to remove MB from industrial water, this approach would provide a new perspective on developing new MB-embedded nano or microparticles for PDT (Figure 4) [59].

As another encapsulation technique, water-soluble MB can be incorporated into NPs. A biodegradable NP composed of dioctyl sodium sulfosuccinate (Aerosol OT^TM^; AOT) and sodium alginate has been utilized to achieve a sustained release of MB [60]. The MB loading efficiency is dependenton both the sodium alginate concentration and the AOT concentration. It is likely that negatively charged sodium alginate helps to entrap the MB while forming a gel-like structure via calcium within the AOT-alginate nanoparticles. In these biodegradable nanoparticles, branched hydrocarbon AOT tails form a bilayer structure while a sulfonic head group of AOT surrounds a calcium-crosslinked alginate matrix. In one study, a zwitterionic polymer-lipid, poly(12-(methacryloyloxy)dodecyl phosphorylcholine), was used to create MB-encapsulated liposomes [61]. The fabricated MB-liposomes exhibited stable liposomal features, including a storage stability of up to 14 days, and more ROS was created by MB-liposome compared to free MB when triggered by six minutes of irradiation with 165 mW of light at 660 nm.

### 2.2. Methylene Blue in Inorganic Nano and Microparticles

**Figure 5:**
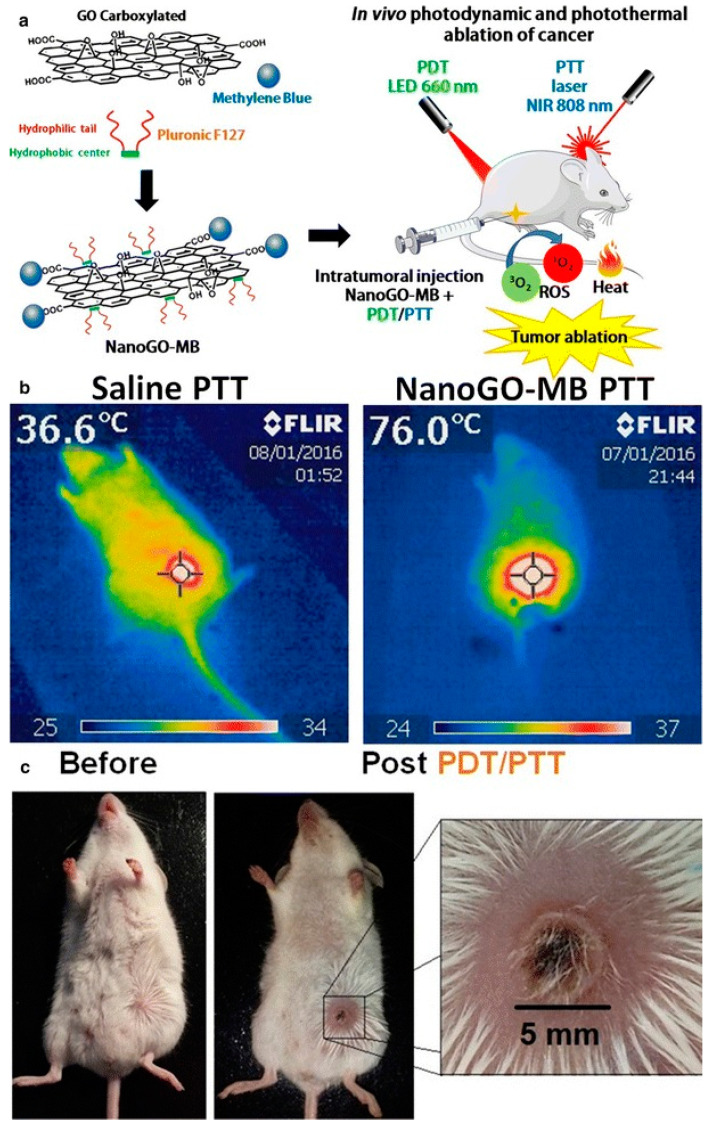
Combined PDT/PTT treatments with nanoGO-MB. (**a**) Schematic representation of nanoGO-MB phototherapies. On both therapies, only the tumor region was exposed to the irradiation, thus the other areas were protected from the light. (**b**) Real-time thermal camera imaging during PTT treatment. A temperature increase of approximately 40 °C was observed only at the tumor region. In this study, the 4T1-Luc tumor-bearing mice were used. (**c**) Tumor site after combined PDT/PTT therapies. The healing tissue measured 5 mm. Reproduced from [55].

In a recent study, MB was successfully incorporated into silica nanoparticles (SNPs), forming nanoparticles with a 190 nm average diameter [52]. The SNP was fabricated using a modified Stöber method without any surfactant in order to load MB molecules into the negatively charged silica. The same work also used organically modified silicate (ORMOSIL) nanoparticles obtained by a two-step method for hollow silica particles using phenyltrimethoxysilane (PTMS) and methyltrimethoxysilane (MTMS) [62]. ORMOSIL is a highly porous inorganic material capable of holding different organic molecules and biomolecules such as oxazine-1, Coumarin-480, and Rhodamine-6G [63,64,65]. The porosity of ORMOSIL-based material ranges between 4 nm and 10 nm, offering microchannels that allows sustained delivery of entrapped molecules [66]. In terms of functionality, gold nanoparticles (GNPs) are a promising carrier for several drugs, including MB. Because of its unique physical, chemical, electrical, and optical properties, GNPs have been extensively utilized as multifunctional nanomedicine platforms [67,68,69,70]. A modified Turkevich method can create citrate-capped gold nanoparticles in order to load MB, which is a positively charged photosensitizer [71]. Citrate forms a physisorbed passivation layer onto the gold nanoparticles that stabilize the gold suspension. The interaction of absorbed citrate molecules can be explained as the physisorbed interaction, because physisorption describes all weak electrostatic interactions including van der Waals interaction [72]. In the formation of the citrate-capped GNPs through this technique, trisodium citrate helps to form 9 to 120 nm GNPs with negatively charged surfaces, which easily interact with positively charged molecules such as MB. Compared to polymeric platforms associated with MB, GNP inorganic matrices can increase the potential of MB for therapeutic benefits, as they have excellent thermo-plasmonic activities [73]. In this regard, combination treatment with MB and GNPs has resulted in enhanced photodynamic inactivation of *Staphylococcus epidermidis*, leading to augmentation of the antimicrobial activity of MB [74]. MB-loaded gold nanoparticles prepared by ultrasonication from the modified Turkevich method reduced clinical *S. aureus* isolates when irradiated by a 660 nm diode laser [54]. MB-GNP was demonstrated to inhibit recalcitrant pathogenic *Candida albicans* fungal biofilm as well [75]. As another inorganic nanomaterial, nano graphene oxide (nanoGO), was also introduced to create an inorganic MB conjugate [55]. Positively charged MBs interact with nanoGO, creating nanoGO-MB, which shows *in-vivo* photodynamic and photothermal ablation of breast cancer (Figure 5).

### 2.3. Methylene Blue in Inorganic Polymeric Composites

Entrapment of MB within polymeric materials may provide better biocompatibility, however, more versatile types of functional materials for MB can be achieved with inorganic polymer composites. In one study, MB was entrapped in silicone elastomers with nanogolds and sustained within the elastomeric polymer composite, since the interaction between the MB and nanogolds would limit the leakage of the highly water-soluble MB over time [76]. Controlled MB delivery platforms using inorganic polymeric composites enables us to expand the range of medical applications for MB, including medical devices for cancers and infectious disease. For example, Noimark et al. incorporated 660 nm laser-activated MB and 2 nm gold nanoparticles into commercial polyvinyl chlorides (PVCs) to inhibit *Staphylococcus epidermidis* and *Escherichia coli* [77]. In a similar work, a mixture of MB and gold nanoparticles were incorporated into a silicone polymer, which is one of the most common biocompatible materials for medical devices [78].

## 3. Methylene Blue in the Photodynamic Therapy

As described previously, PDT uses a photosensitizer (PS) that creates ROS when activated by a specific light source. This easy-to-use clinical modality has been used in the treatment of superficial skin cancers as well as acne. Additionally, PDT can be adopted either as an independent modality or in combination with another regimen to improve clinical outcomes in cancer treatment. There have been a number of clinically approved PDT protocols for the treatment of various cancers, including skin, bladder, lung, esophagus, and cervix cancer [27]. The engulfed PS within cancer cells is excited enough to generate intercellular ROS, leading to tumor cell death. A comprehensive review on PDT for cancer treatment demonstrated that it involves all of the three major tumor-regression pathways: cell death, damage of microvasculature, and induced inflammatory reactions [16].

### 3.1. In-Vitro Observation of Methylene Blue (MB)-Mediated PDT in Cancer Treatment

Several studies have aimed to elucidate the mechanism of MB-mediated PDT. One study used radiolabeled ^125^I- and ^211^At-methylene blues to demonstrate that MB can be selectively concentrated in pigmented melanoma, indicating that MB has an affinity to melanin [79]. Another study indicated that mitochondria is the intercellular binding site where photoactivated MB creates singlet oxygen, leading to mitochondrial damage and subsequent apoptosis [80,81]. Proteomic analysis of expressed proteins from treated B16F1 melanoma cells revealed that MB-induced ROS is responsible for the altered expression of mitochondrial proteins, leading to apoptosis via caspase-3/9 apoptosis pathways [81]. MB, which is one of the phenothiazines that efficiently absorb light, has been recognized for treating cancer cell lines.

A growing number of in-vitro studies with MB indicate that the photosensitizer has great potential for selectively treating cancer cells. Mouse Gardner lymphoma, sarcoma 180, Ehrlich ascites, and mammary adenocarcinoma have all been successfully treated using MB in animal models [82]. Likewise, some human cancer cells have been reported as responders of MB-mediated light therapy, including HeLa cervical adenocarcinoma cells, early stage head and neck squamous cell carcinoma (HNSCC), and lung adenocarcinoma cells [83,84,85]. Although some studies have indicated that the cytotoxicity of MB can be improved by chemical modifications, these studies are beyond the scope of this review, particularly considering the functionality of the safe, proven molecules in the treatment of a variety of cancers using MB-mediated light therapy. One recent study demonstrated the potential of MB-mediated light therapy to inhibit human breast cancer cells *in vitro*. This study showed that MB-mediated PDT inhibits the growth of MDA-MB-231 cells, which are Triple-Negative Breast Cancer (TNBC) cells [86]. Only 20% of breast cancers are TNBC, meaning they do not express estrogen receptor, progesterone receptors, or human epidermal growth factor receptor-2. With the application of MB-mediated light therapy, the intercellular level of reduced glutathione (GSH) remains low, comparable to normal conditions. It was speculated that the innate nature of reducing GSH may contribute to the rapid impact of oxidative stress. Another study found that treatment with antioxidant in conjunction with PDT reduces ROS production, thereby negating the effect of MB-mediated PDT. Hence, combination treatment with both MB and PDT increase ROS-induced cell death.

Similarly, osteosarcoma is another subject for MB-mediated PDT. Two studies showed that MB is responsible for inducing necrosis and apoptosis of mouse osteosarcoma (LM8) and osteosarcoma-derived UMR 106 cells [87,88]. Both studies also demonstrated that the source of light does not affect the efficacy of this convenient therapeutic modality. Since MB is more toxic to cancerous cells than non-cancerous cells, this photosensitizer is a great option for improving the long-term use of PDT. Treatment of early-stage head and neck squamous cell carcinoma (HNSCC) is another example of a MB-mediated PDT application. Kofler et al. showed the effect of MB-mediated PDT activated with a diode laser (660 nm) on HNSCC [84]. The authors demonstrated that MB is an effective photosensitizer for reducing HNSCC, which is usually difficult to treat without surgical interventions. They also noted that dark cytotoxicity of MB should be considered in treating cancers. To maximize the efficacy of this treatment, three parameters warrant consideration: concentration of MB, time of light exposure, and dark toxicity that can be shown by photosensitizers.

### 3.2. In-Vitro and In-Vivo Applications of Methylene Blue (MB)-Mediated Antimicrobial Photodyanmic Therapy (aPDT)

**Figure 6:**
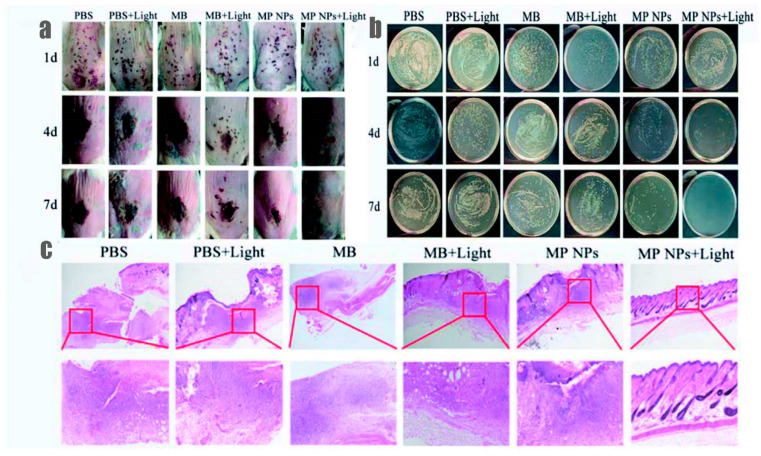
(**a**) Photographs of infected wounds in mice at different timepoints after different treatments: PBS, PBS + light, MB, MB + light, MPNPs, and MPNPs + light. (**b**) Photographs of bacterial colonies formation on LB agar plates from the infected wounds after different treatments. (**c**) H&E staining (Hematoxylin and Eosin) of tissue sections of MRSA infected wounds in mice with different treatments. The scale bar is 500 μm [94].

MB was among the first generation of photosensitizers to demonstrate antimicrobial activity with light activation (Table 2) [89,90]. According to this seminal observation, MB facilitates the rapid killing of paramecia, which are unicellular ciliates easily found in freshwater, when exposed to normal daylight. In the dark, however, MB did not influence the survival of paramecia. In antimicrobial photodynamic therapy (*a*PDT), light-activated MB, which likewise stimulates ROS generation, is likely to inhibit microbial activity, even within bacterial biofilms, expediting the eradication of microbial infection. Many previously published studies showing the effect of *a*PDT on bacterial inhibitions, either alone or in combination with antibiotics, suggest that MB-mediated *a*PDT may be an alternative therapeutic option for reducing the misuse of antibiotics [90]. In fact, antibiotic overuse has become one of the top medical problems in the modern age, and has contributed to the emergence of multiple-drug-resistant bacteria [91]. *a*PDT may be an optional therapy to overcome bacterial resistance. In this review, we have highlighted several in-vitro and in-vivo studies of MB-mediated *a*PDT in human infectious diseases. Based on the advantages of *a*PDT, there have been attempts to use MB-mediated *a*PDT for treating infectious bacteria, including *Staphylococcus aureus* and *Pseudomonas aeruginosa* [92,93]. In one study, lactic-*co*-glycolic acid (PLGA)-coated M B nanoparticles (MPNPs) were evaluated for the treatment of methicillin-resistant *Staphylococcus aureus* (MRSA) infections in-vivo where the MRSA infected mice were irradiated with a 660 nm laser at a power density of 1 W/cm^2^ for 5 min. Compared to the control group over seven days, the MPNPs plus light treatment group showed a significant wound reduction associated with eradicating MRSA, which was confirmed by observing the colony-forming unit (CFU) results from each treatment at each time point (Figure 6) [94].

Multiple bacterial infections localized in specific parts of the body cause a variety of diseases. For example, both methicillin-resistant *S. aureus* (MRSA) and *P. aeruginosa* are commonly found in chronic diabetic wounds and are recognized as inhibiting wound healing [95]. Dental caries is also attributed to bacterial infection of the tooth, and *Streptococcus mutans* is indicated as a major cause of dental decay [96]. MB-mediated *a*PDT has been utilized to remove or inhibit the cause of these diseases. For treating in vitro dentin caries microcosms, a MB solution and a red LED light source with different energy densities are used [97]. When applied on the in vitro model of dentin caries microcosms, 100 mg/L of MB solution and 75 J cm^−2^ LED irradiation exhibited the best result in reducing associated bacteria, including *S. mutans*. Almost 23.9% of *S. mutans* was eradicated through this single treatment. In another in vitro study, *S. mutans* biofilms were treated with 100 µM MB under 660 nm-LED irradiations with different doses. *S. mutans* biofilms were inactivated after several minutes of treatment, indicating the potential of MB-mediated aPDT for the treatment of dental caries [98]. MB-mediated *a*PDT can also be used for peri-implantitis therapy. The oral biofilm that grows on the surface of implants can result in damage to and ultimate failure of dental implants. Gram-negative *Porphyromonas gingivalis* and *Aggregatibacter actinomycetemcomitans* and Gram-positive *S. mutans* were seeded onto sandblasting, large-grit, and acid-etching (SLA)-pretreated titanium alloy implants and subsequently subjected to MB-mediated *a*PDT in vitro [99]. Although the authors observed the best killing efficacy of those bacteria in the harshest conditions, including a pH of 10 and 200 µg/mL MB, this study indicates the potential of the MB-mediated *a*PDT as a treatment for peri-implantitis.

## 4. Future Perspective on Methylene Blue Fabrications

To improve the bioavailability of MB for PDT, development of efficient MB-loaded drug delivery systems is of great importance. While improving the therapeutic efficacy of MB associated with PDT, the nano and microparticles should show sustained and localized MB distribution near the target sites. In this regard, a high-MB loaded liposomal formulation would be a good candidate for the sustained and localized delivery of MB, creating an attractive methylene blue-based vesicle that offers better translatable PDT modalities in various disease treatments [100]. MB enclosed within the hydrophobic and biocompatible lipidic materials of the liposome will prevent the rapid loss of drug in the PDT-based treatment, as the drug will be slowly released into the area [101]. In addition, customizable features of a liposomal formulation enables us to create an on-demand PDT modality. For example, cationic liposomes were successfully used to enhance the permeability of MB within the biofilms, which is one of the critical factors leading to antibiotic resistance [102]. Similarly, MB could be internalized within aqueous core nanocapsules created by a thin polymer layer [103]. Compared to conventional nanoparticles made from polymers, the aqueous core nanocapsules are able to hold hydrophilic molecules with a relatively small amount of polymer for a tunable pharmacokinetic profile and bioavailability. Through higher MB loadable vesicles fabricated by the aforementioned techniques, PDT can become a more effective and more viable option for the treatment of cancers and infectious diseases.

## 5. Conclusions

MB has been utilized as a photosensitizer for PDT in the treatment of cancers and infectious diseases. Despite the early use of this hydrophilic molecule in PDT, few studies have been reported to improve MB has been utilized as a photosensitizer for PDT in the treatment of cancers and infectious diseases. Despite the early use of this hydrophilic molecule in PDT, few studies have been reported to improve the loading capacity of MB. It has been speculated that the hydrophilic nature of MB might limit the clinical efficacy of MB-mediated PDT. As reviewed here, however, recent technological development in creating nano and microparticles for targeted drug delivery is likely to increase the chance of the presence of MB in the target area. A polymeric micro composite complexed with MB shows the formation of singlet oxygens for cancer cell treatment. Similarly, coating onto the surface of a gold nanoparticle, covalent attachment onto the surface of a functionalized graphene, and co-formation of a silica hollow nanoparticle are good examples of MB encapsulation techniques to create a successful MB delivery system for the enhanced MB-mediated PDT. These functional materials show the potential benefit of delivering MB to a disease area of interest and provide insight into how MB-carrying materials help increase the clinical efficacy of PDT. Moreover, recently studied techniques such as liposome and aqueous core nanocapsules offer the promising potential of MB-mediated PDT, where MB can localize in the specific target area and be a safe photosensitizer for treating a wide range of cancers and infectious diseases in the near future.

## Figures and Tables

**Table 1:** Examples of clinical use of methylene blue (MB).

Indications	Descriptions	Ref.
Alzheimer’s disease	Positive effects are proposed through multiple neurological systems such as cholinergic, serotonergic and glutamatergic neurotransmitter systems MB reduces aggregated amyloid-β (Aβ) peptide while preventing tau aggregation A recommended dose of 3 × 60 mg/day for treatment	[11,34,35,36,37]
Hepatopulmonary Syndrome	Intravenous dose of MB (3 mg per kg) for treatment	[38,39,40]
Ifosfamide-induced encephalopahty	Intravenous dose of MB (6 × 50 mg/day) for treatment	[41,42,43]
Malaria	MB has the high antimalarial potency (IC50 = 4 nM) against *Plasmodium falciparum* MB inhibits *P. falciparum* glutathione reductase (P*f*GR) known as a drug target against Malaria MB is a partner drug for combination therapies	[2,44,45,46]
Methemoglobinemia	MB is an electron donor for the non-enzymatic reduction of methemoglobin	[47,48,49] ^1^

^1^ The effect of MB on Methemoglobin can be reversed, for example, in aniline-induced methemoglobinemia [50].

**Table 2:** Phototoxicity of methylene blue against typical types of pathogenic bacteria ^1^.

Strains	Minimum Lethal Concentration (µM)		Type of Bacteria
	Dark	Light	
*Staphylococcus aureus*	2.5	1	Gram-negative
*Enterococcus faecalis*	500	100	
*Bacillus cereus*	1000	1000	
*Escherichia coli*	100	100	Gram-positive
*Pseudomonas aeruginosa*	500	125	

^1^ The experiments were performed by a non-laser light source at a fluence of 1.75 mW/cm^2^. A total light dose was 6.3 J/cm^2^ for 1 h illumination [90].

## Data Availability

Not applicable.
